# Risk factors associated with the occurrence of *Brucella canis* seropositivity in dogs within selected provinces of South Africa

**DOI:** 10.4102/jsava.v90i0.1956

**Published:** 2019-09-25

**Authors:** Johan Oosthuizen, James W. Oguttu, Charne Etsebeth, Werner F. Gouws, Folorunso O. Fasina

**Affiliations:** 1 Department of Agriculture and Animal Health, College of Agriculture and Environmental Sciences, University of South Africa, Johannesburg, South Africa; 2 Western Cape Provincial Department of Agriculture, Riviersonderend, South Africa; 3 Department of Veterinary Tropical Diseases, University of Pretoria, South Africa; 4 Emergency Centre for Transboundary Animal Diseases, Food and Agriculture Organization of the United Nations, Dar es Salaam, Tanzania

**Keywords:** zoonotic disease, *Brucella canis*, tube agglutination test, abortions, dog age, risk factors, dog ownership

## Abstract

The growing population of free-roaming dogs in informal communities in South Africa may increasingly place humans at risk of possible zoonotic infections including, but not limited to, *Brucella canis*. Worldwide, the prevalence of *B. canis* infection has increased during the last two centuries, resulting in increased reports of dog and human infections. This study investigated the risk factors associated with *B. canis* infection in dogs in three predefined areas: Gauteng, the Eastern Cape and Western Cape provinces, of South Africa. Dogs aged 7 months and older presented to welfare organisations and breeders in the study areas were selected for sampling. A comprehensive questionnaire on dog ownership, general health and vaccination status was completed prior to sampling. One blood sample of 8 mL was collected aseptically per dog. Then, equal amounts (4 mL) were transferred to the different vacutainer tubes. The 2-mercaptoethanol-tube agglutination tests were used after validation. Fifty-two dogs out of the combined sample of 1191 dogs from the three study areas tested positive for *B. canis*, representing an overall occurrence of 4.4%. A binomial logistic regression model was fitted to identify risk factors associated with *B. canis* in dogs within the study areas. Dog age (0.371; *p* < 0.05) and external parasite infestation (0.311; *p* < 0.05) were significantly associated with the *B. canis* infection. Ownership and sterilisation need to be further investigated as possible risk factors because both had odds ratios of 1684 and 1107, respectively, in the univariate model.

## Introduction

For thousands of years, dogs have been people’s closest friend and companion, and depended almost entirely on humanity for food, shelter and care (Wang et al. 2016). The emergence of rural and peri-urban slums and informal communities has resulted in increased urban and peri-urban dog populations and scavengers (Marzetti et al. 2013). The presence of stray and uncontrolled dogs originating from different locations in South Africa and possibly from neighbouring countries, together with poor levels of biosecurity, hygiene and relative lack of infrastructure in informal settlements, facilitates infection of dogs with pathogens, including *Brucella* spp.

Katona and Katona-Apte (2008) noted that malnutrition causes immunodeficiency worldwide, with groups such as infants, children, adolescents and the elderly most affected, emphasising the close relationship between malnutrition and primary infections in immune-compromised humans. Mason, Musgrove and Habicht (2003) indicated that there is a possibility to reduce the world’s disease burden by at least 32% if poverty and malnutrition are addressed. The burden of poverty is complicated by HIV and AIDS infections in poorer communities, with a resultant increase in the numbers of immune-compromised humans, which can be aggravated by concurrent infection with *Brucella canis*.

According to the 2011 statistics of the City of Johannesburg Municipality, Gauteng (CJM-Gauteng), 124 075 informal dwellings (shacks in a backyard) and 125 748 informal dwellings (shacks not in a backyard but in an informal settlement or on a farm) exist around Johannesburg, and a huge number of children under the age of 18 live in informal dwellings (Statistics South Africa 2013). Based on the Johannesburg Community Survey conducted in 2007, 48% of households in informal dwellings have one or more children. The high number of children living in shacks points to the increased risk of exposure to possible infection with zoonotic diseases from stray or pet dogs. Other contributory factors to the increased risk of zoonotic infection from dogs might be the low level of schooling among adults in these communities who may not be aware of the existence of zoonotic diseases. According to the Housing Development Agency (2013), the proportions of dwellings classified as informal settlements have gradually declined, from approximately 20% in 2001 to 9% of the total households by 2011, in the Nelson Mandela Bay Municipality, Eastern Cape. Informal dwellings in the Overstrand and Theewaterskloof municipalities in the Western Cape made up between 16.4% and 17.5% of total households as in 2014 (WCGPT 2015).

Lucero et al. (2005) are of the view that human cases of infection with *B. canis* are probably underreported and could be more common than what is indicated in published reports. This is attributed to the difficulty of diagnosing *B. canis* infection in humans. Furthermore, Lucero et al. (2010) suggest that the risk of *B. canis* infection for people handling dogs or in close contact with dogs, especially dogs kept in kennels, was higher than in people who do not come into close contact with dogs.

In the majority of reported human cases, *B. canis* infection is the direct result of exposure to whelping females when high concentrations of the *Brucella* organisms occur in the birth fluids and vaginal discharge (Kazmierchak 2012). The most common symptoms reported in humans (particularly young children) infected with *B. canis* include fever, diarrhoea, vomiting, headache, fatigue, myalgia and nausea as well as clinical signs of endocarditis (Nomura et al. 2010). Co-habitation with dogs significantly heightens the risk of *B. canis* infection among mates, with urine being the most important source of infection.

Since the first report of *B. canis* in dogs in the United States (US) in 1966, the bacterium has been detected globally, presenting itself in various forms. *Brucella canis* has now been reported in the US, Canada, Central and South America, some European countries, Tunisia, Nigeria, South Africa, Madagascar, the Philippines, Malaysia, India, Korea, Japan, Taiwan and China (CFSPH 2018).

The risk factors and prevalence of *B. canis* in dogs and humans in South Africa to date are unknown, with only a few positive cases in dogs reported and no relevant targeted study has been conducted. Gous et al. (2005) believed that the two cases found in dogs in the Western Cape province, 5 months apart, might have been indicative of a higher prevalence of *B. canis* in South Africa than that recognised at the time. They proposed that *B. canis* might also be endemic in South Africa, with distribution possibly limited to the stray dog population in informal settlements (Gous et al. 2005).

Although cases of *B. canis* in dogs in South Africa have previously been reported by Van Helden (2012) in the towns of Bedford, Somerset West, Hermanus and Knysna (Gous et al. 2005), this is the first study in South Africa that investigated the occurrence and risk factors of *B. canis* in dogs in multiple provinces of South Africa. The need for a study such as this one is underscored by the fact that notwithstanding the reported isolated cases of *B. canis* in dogs, no study to date has investigated the occurrence of *B. canis* in humans in South Africa.

Therefore, the objective of this study was to investigate the risk factors associated with the occurrence of *B. canis* among dogs originating from different communities in three selected provinces of South Africa to provide empirical evidence to guide informed decisions on the future control and prevention of *B. canis* by the regulatory authorities.

## Materials and methods

The study was conducted in selected locations of the City of Johannesburg Municipality-Gauteng (CJM-Gauteng), the Port Elizabeth Municipality in Nelson Mandela Bay Metropolitan (NMBM), the Eastern Cape province and Theewaterskloof and Overstrand (T&O) municipalities in the Western Cape province, South Africa. The CJM-Gauteng, including all its surrounding cities and districts, is by far the biggest metropolitan area in the country, boosted significantly by the wide net of smaller cities that constitute the megacity. According to the City of Johannesburg 2011 census, the City of Johannesburg Local Municipality has a total population of 4.4 million of which 76.4% are black Africans, 12.3% are white people, 5.6% are mixed race people and 4.9% are Indians or Asians. There are 1 434 856 households in the municipality with an average household size of 2.8 persons per household. As there is a provision for a maximum of two dogs per household (City of Johannesburg 2011; Statistics South Africa 2013), the total maximum number of dogs expected in the study area is 284 106 × 2 = 568 212 dogs.

In NMBM, the study was conducted predominantly within the greater Port Elizabeth area, Eastern Cape, in collaboration with the state veterinarian and welfare organisations situated in the townships. The NMBM covers an area of 1958.91 km², with Port Elizabeth, one of the largest cities in South Africa, being located within the NMBM and the Algoa Bay region (ECSECC 2010). According to the Nelson Mandela Bay Integrated Development Plan 2011–2016, the metropole has a current population of 1.3 million, making it the fourth largest city in South Africa.

The Western Cape part of the study was conducted in two local municipalities in the Overberg region of the Western Cape, namely, the T&O municipalities. They cover an area of 4940 km^2^ in the western interior of the Overberg region. Theewaterskloof has a total population of 117 167 (2016) and a total number of 33 118 households, 77.5% of which reside in permanent dwellings and approximately 7500 households staying in informal dwellings occupying the area between the Riviersonderend mountains to the north and the Kogelberg and Kleinrivier mountains to the south. The Overstrand Local Municipality (OLM) is the smallest of the four municipalities, which stretches over only 675 km² and has a total population of 93 407 and 35 718 households with an average household size of 2.6 persons per household.

During the presentation of dogs for welfare services or treatments, dogs were recruited into the study using the simple random sampling method whereby each member of the subset has an equal probability of being chosen from among the group. This method was accepted because the total population of dogs to be sampled was unknown in each location. Dogs older than 7 months were taken as mature and thus sexually active.

Determination of the sample size was based on population estimates of 11.1 humans per dog ratio for rural areas (Rautenbach, Boomker & De Villiers 1991), and the fact that a sample size for a large population or theoretically infinite population is 323, estimated with a desired absolute precision of 5% with a confidence interval of 95%. Based on these guidelines, a sample size of 400 was used for the Western Cape, Eastern Cape and CJM-Gauteng.

A pretested three-part structured questionnaire was prepared and completed by each participant. The first section of the questionnaire comprised demographic details of the dog owners. The second section of the questionnaire comprised the information about the dog, such as the breed, dog size, geographic location, parasite burden and body condition of the animal. Body condition scoring (BCS) was performed using the five-point Hill’s BCS method (Anon 2010). In the third section, all clinical signs associated with *B. canis* were collected. In addition, vaccination history and sterilisation information was recorded. Detailed information regarding the history of origin of the animal was recorded for future reference.

Serum and heparinised blood samples (one sample from each dog) (*n* = 1191) were collected aseptically using an 8 mL syringe and 0.5 inch precision glide 20-gauge needle from the dog’s cephalic vein. Each blood sample was divided on site equally into a serum tube without coagulant and a tube containing heparin. All blood samples were marked with a pre-allocated dog number, packed in a cooler box (≈4 °C) and transported to the University of South Africa (UNISA) Bacteriology Laboratory at the Florida Science Campus, the State Veterinary Office in Port Elizabeth or the Stellenbosch Veterinary Laboratory (SVL). At the laboratory, sterile blood samples from the red top vacutainer were left for at least 3 h to ensure that clotting had taken place before being centrifuged at 2800 rpm for 15 min. The serum was then transferred, using a pipette, to a pre-marked 2 mL cryotube for overnight storage at −20 °C. Similarly, the heparinised blood samples were transferred directly into 2 mL pre-marked cryotube containers, refrigerated overnight at −4 °C. The two samples were packed in sterile containers, sealed and marked before being transported according to the World Organization for Animal Health (Office International des Epizooties [OIE]) protocol for transporting infectious agents (OIE 2012), to the ARC-OVR in Pretoria for analysis.

The 2-mercaptoethanol-tube agglutination test (2ME-TAT), recommended by the Department of Agriculture Forestry and Fisheries (DAFF), was first validated and then employed to test the samples for the presence of antibodies among positive reactors. For the determination of diagnostic sensitivity, the following samples were used: 3 reference sera and 28 proficiency test samples. From the results obtained, the sensitivity was found to be 100% (31/31). For the determination of diagnostic specificity, the following samples were used: 1 reference serum and 12 proficiency test samples. From the results obtained, the specificity was found to be 100% (13/13). For the determination of repeatability, two positive reference sera, one negative reference serum, one Synbiotics kit positive control sample and five proficiency samples were tested on three occasions and there was 100% agreement for all results. The 2ME-TAT was performed at the ARC-OVR bacteriological and serological laboratory with the following serial number: # 17-1202; the controls (high positive, medium positive and negative controls) had the following serial numbers: # 212-H 0601 (high), # 212-M 0402 (medium) and 212-N 0402 (negative).

Culture of positive reactors was performed at the SVL. All negative cultures were sub-cultured at intervals to confirm the presence or absence of any bacteria. A positive *B. canis* was used as a control.

The data were analysed using IBM SPSS Statistics version 22. Descriptive statistics were summarised and presented as frequencies, percentages and means. Pearson’s chi-square test was used to examine associations between categorical explanatory variables and the outcome variable (disease status of the dog). In cases where the variables involved exceeded two categories, pairwise comparisons were performed and the Bonferroni method was used to adjust the *p*-value and lower the chance of making Type 1 errors (Sedgwick 2012). For continuous explanatory variables such as BCS, the one-way analysis of variance (ANOVA) method was used and, where associations were found, Tukey’s honestly significant difference (HSD) test was performed to identify exactly where the statistically significant differences existed.

A logistic regression model was fitted to the data to identify risk factors associated with *B. canis* in dogs within the study areas. Variables for inclusion in the multivariable logistic regression were determined by first fitting the univariable model. A less strict *p*-value of 0.25 was set as the cut-off point for selection of variables from the univariable analysis to include in the multivariable model, as the more commonly used level of 0.05 can exclude variables that are known to be important.

### Ethical considerations

Ethical clearance to conduct this study was obtained in 2014 from the University of South Africa, College of Agriculture and Environmental Sciences ethics committee before the study commenced (Ethical clearance number: 2014/CAES/061). In addition, Section 20 approval to conduct this study was also obtained from the Department of Agriculture, Fisheries and Forestry (DAFF).

## Results

The analysis was based on a sample of dogs (*n* = 1191) drawn from the three study areas (Table 1). The overall mean age of the 1191 dogs sampled was 38 months. However, there were notable differences in the average age of dogs, with CJM-Gauteng having the youngest mean age of 29.9 months compared to 43.4 months for NMBM and 40.6 months for T&O (Table 1).

**TABLE 1:** The proportion of *Brucella canis* positive cases in the study area.

Variable	Mean (SD)	Sample (*N*)	Positive cases	
			Numbers (*n*)	Proportions (%)
**Study area**				
CJM-Gauteng	-	391	5	1.3
NMBM	-	400	39	9.8
T&O	-	400	8	2.0
**Mean age (months)**				
CJM-Gauteng	29.9	391	NA	NA
NMBM	43.4	400	NA	NA
T&O	40.6	400	NA	NA
**Sex**				
Female	-	734	31	4.2
Male	-	457	21	4.6
**Body size**				
Small	-	265	7	2.6
Medium	-	612	38	6.2
Large	-	314	7	2.2
**Dog ownership**				
Household	-	858	44	5.1
Stray	-	333	8	2.4
**Sterilisation**				
Sterilised	-	366	15	4.1
Not sterilised	-	820	37	4.5
Uncertain	-	5	-	-
**BCS**				
Good	-	224	7	3.1
Acceptable	-	746	32	4.3
Poor	-	221	13	5.9
**Presence of external parasites**				
None	-	607	12	2.0
Slight	-	469	33	7.0
Mild	-	79	3	3.8
Severe	-	36	4	11.1
**Presence of clinical signs**				
None	-	1059	45	4.2
Repro	-	60	5	8.3
Joint	-	24	1	4.2
Other	-	48	1	2.1
**Total**	**-**	**1191**	**52**	**-**

Note: Vaccination history was inadvertently missed out in the NMBM data set, and hence the decision to exclude the NMBM data on vaccination history.

NA, not applicable; SD, standard deviation; CJM, City of Johannesburg Municipality; NMBM, Nelson Mandela Bay Metropolitan; T&O, Theewaterskloof and Overstrand municipalities; BCS, body condition scoring.

Fifty-two dogs out of the combined sample of 1191 dogs from the three study areas tested positive for *B. canis*, representing an overall occurrence of 4.4%. The proportion of positive cases was highest in the NMBM study area (9.8%; *n* = 39/400), compared to T&O (2.0%; *n* = 8/400) and CJM-Gauteng (1.3%; *n* = 5/391) (Table 1). Fewer than 5% (*n* = 52/1191) of dogs from all the three study areas tested positive for *B. canis*, representing an overall crude prevalence of 4.4%. The percentage of dogs that tested positive for *B. canis* was 5.1% (*n* = 44/858) among household dogs, compared to 2.4% (*n* = 8/333) among stray dogs. The chi-square test results showed that *B. canis* occurrence was significantly higher among household dogs compared to stray dogs (chi-square = 4.010, *p* = 0.04). Likewise, infection with parasites was significantly associated (chi-square = 279.459, *p* = 0.00) with the occurrence of *B. canis* in dogs, with dogs that had severe infestation having a higher proportion of positive cases (11.1%) compared to those with slight infestation (7.0%), mild infestation (3.8%) and no infestation (2.0%).

Both male and female dogs had almost similar levels of occurrence (4.6% vs. 4.2%). Medium-sized dogs had a higher occurrence (6.2%) compared to small dogs (2.6%) and large dogs (2.2%). Household dogs had a higher occurrence (5.1%) compared to stray dogs (2.4%). Sterilisation status appeared not to influence the occurrence of *B. canis* (Table 1). Dogs with poor body conditions had a higher proportion of *B. canis* infection (5.9%) compared to others, and dogs with reproductive conditions (9.3%) had a higher positive number compared to those with joint illnesses (4.3%), no conditions (4.2%) and other conditions (1.9%).

Information pertaining to dog ownership revealed that overall 72% (*n* = 858/1191) of the dogs sampled had traceable owners, while 28% (*n* = 333/1191) were classified as stray dogs (Table 1). The percentage of stray dogs was highest in T&O (42%; *n* = 168/400) and lowest in NMBM (12%; *n* = 48/400). Out of a total of 1191 dogs from the three study areas, the majority (69%; *n* = 820/1191) were not sterilised, thus only 31% (*n* = 366/1191) were sterilised. Only a very small number (0.4%; *n* = 5/1191) were classified as uncertain (Table 1).

From the three study areas, 62.6% (*n* = 746/1191) of the dogs were classified as having an acceptable BCS (score of 3), while there were almost equal proportions for BCS 4 or 5 (18.8%; *n* = 224/1191). Dogs with a BCS considered to be poor (one or two) were the lowest in number (18.6%; *n* = 221/1191) BCS (Table 1).

Altogether most dogs (89%; *n* = 1059/1191) included in the study did not show any clinical signs, 5% (*n* = 60/1191) showed signs of reproduction problem-related clinical signs, joint problem signs (2%; *n* = 24/1191) and other related signs (4%; *n* = 48/1191).

About half (51%; *n* = 607/1191) of the dogs from the three study areas did not have any external parasites. However, 39% (*n* = 469/1191) of the dogs had a slight infestation, followed by 7% (*n* = 79/1191) that had mild infestation. Only 3% (*n* = 36/1191) were severely infested with external parasites.

Based on the initial univariable analysis (Table 2), variables that were shown to be significant predictors of *B. canis* on the generous Wald chi-square statistic (*p* ≤ 0.25) were selected for inclusion in the multivariable model and these were age (Wald chi-square = 15.113, *p* = 0.00), external parasites (Wald chi-square = 14.725, *p* = 0.00) and ownership (Wald chi-square = 3.834, *p* = 0.05) (Table 2). The other three variables that included clinical signs, sex and sterilisation fell short of the cut-off *p*-value of 0.25 and therefore were excluded from the next step.

**TABLE 2:** Wald’s chi-square results from the univariable logistic regression models.

Variables	B	SE	Wald	Df	Sig.	Exp (B)
Age	−1.119	0.288	15.113	1	0.000*	0.326
Clinical signs	−0.236	0.417	0.319	1	0.572	0.790
External parasites	−1.283	0.334	14.725	1	0.000*	0.277
Ownership	0.764	0.390	3.834	1	0.050*	2.146
Sex	−0.083	0.289	0.083	1	0.773	0.920
Sterilisation	0.102	0.313	0.106	1	0.745	1.107

Note: Data set in bold are the variables found to be of higher risk.

B, Bonferroni correction; SE, standard error; Df, degrees of freedom; Sig, significance; Exp (B), expressions.

* , *p* was set at ≤ 0.25 for the univariable analysis.

The three significant categorical variables – age, external parasites and ownership – had a significant influence on *B. canis* and were thus entered as covariates in a multivariable binomial logistic model. These three predictor variables were labelled or coded as follows: (1) age: 7–36 months (*n* = 806), older than 36 months (*n* = 385); (2) external parasites: none (*n* = 607), yes (*n* = 584); (3) ownership: household (*n* = 858), stray (*n* = 333) for analysis.

The model was checked for significance and was statistically significant (chi-square = 31.11, *p* = 0.00). The Nagelkerke *R*^2^ indicated that the model explained 8.6% of the variance in the occurrence of *B. canis* in dogs. The Hosmer–Lemeshow test was used to determine whether the model fitted the data well. This is the case if the indicated *p*-value is more than 0.05 and the model was therefore a good fit (chi-square = 1.970, *p* = 0.85). The classification statistics estimated the logistic regression model to give an accurate prediction on *B. canis* outcomes about 96% of the time.

The contribution of each of the three predictor variables to the model was computed. Ownership (Wald chi-square = 1.727, *p* = 0.19) did not contribute significantly to the prediction of *B. canis* among the dogs, while age and external parasites were both shown to be significant risk factors (*p* < 0.05) (Appendix 1). Because the ownership variable (categorised as household dogs against stray dogs) was indicated as ‘not a significant contributor’ in the model, it was removed from the list of covariates and the logistic model was re-computed. The final re-run model remained significant (chi-square 29.198, *p* = 0.00) and the Hosmer–Lemeshow test showed a good fit (*p* = 0.99). The Nagelkerke *R*^2^ value of 0.080 also remained essentially unchanged, indicating that the model explained about 8% of the variance in *B. canis* among the dogs (Table 3). The two variables retained in the final model were age and external parasites, as shown in Table 3. The Exp (B) coefficient represents the adjusted odds ratio and estimates the change in the odds of *B. canis* positive results in the dogs for a unit change in the respective predictor variable.

**TABLE 3:** Variables in the final binomial multivariable logistic regression model.

Variable	B	SE	Wald	df	Sig.	Exp (B)
Age	−0.992	0.291	11.628	1	0.001*	0.371
External parasites	−1.168	0.337	11.999	1	0.001*	0.311
Constant	−2.096	0.204	105.426	1	0.000	0.123

B, Bonferroni correction; SE, standard error; Df, degrees of freedom; Sig, significance; Exp (B), expressions.

* , *p* < 0.05.

The age of the dog was a significant risk factor for the occurrence of *B. canis* (Wald’s chi-square = 11.628, *p* = 0.00). The indicated Exp (B) value of 0.371 shows that dogs aged 36 months or younger had 63% less risk of testing positive for *B. canis*. Conversely, this means that dogs older than 36 months were 2.7 times more likely to test positive for *B. canis.* External parasites were a significant risk factor for *B. canis* (Wald’s chi-square = 11.999, *p* = 0.00). The indicated Exp (B) value of 0.311 implies that dogs with no external parasites had 69% less risk of testing positive for *B. canis.* Conversely, dogs with external parasites were 3.2 times more likely to test positive for *B. canis* (Table 3).

## Discussion

In this study, we have described the occurrence and risk factors for the detection of *B. canis* in selected provinces in South Africa. Six risk factors were tested: the age of dogs, presence of external parasites, ownership of dogs recruited into the study, clinical signs at the time of presentation, sex of dogs and sterilisation status. Two variables were retained in the final model – age and external parasites – and the other variables were dropped as insignificant.

Sexual activity and predominance are found to be associated with older dogs (Trisko & Smuts 2015) and in a situation where dogs mate with infected dogs, they carry the risk of becoming infected with *B. canis*. Because older dogs also cover a larger roaming area, the risk of environmental contamination from mating and coming into contact with infectious material over a wider spatial dimension may aggravate the occurrence in older dogs.

Globally, the relationship between *B. canis* infection and age has been established in other studies. In Iran, a serological survey for *B. canis* conducted among 102 companion dogs in Ahvaz during 2006–2008 revealed that the group of dogs older than 5 years had a *B. canis* prevalence of 9.3% compared to 1.69% in the younger group (Mosallanejad et al. 2009). Although the difference was not significant, it clearly indicated that higher age is associated with the probability of infection. According to Alfattli (2016), the first documented study of *B. canis* in Iraq revealed the differences in prevalence for *B. canis* in dogs using three different tests: the rapid test kit, indirect enzyme-linked immunosorbent assay (ELISA) and 16S rDNA inter-spacer polymerase chain reaction PCR techniques. They observed 4.8% for dogs < 1 year, 5.36% for dogs between 1 and 4 years and 11.48% for dogs > 4 years of age.

A serological study by Ayoola et al. (2016) between 2011 and 2014 in the Lagos and Ogun states of Nigeria among hunting and stray dogs similarly revealed age to be a significant factor playing an important role in *B. canis* infection. The study found that dogs older than 3 years were more than six times more likely to be seropositive for antibodies for *B. canis* than dogs younger than 3 years. The reason for this as explained earlier is that older dogs are more likely to get exposed to infected material and/or other infected dogs for a longer period, and hence they have an increased risk of getting infected.

Talukder, Samad and Rahman (2012) conducted a sero-prevalence study on 30 stray dogs from the Mymensingh Municipal Corporation area in Bangladesh. The study revealed that the prevalence of *B. canis* in younger dogs up to 7 months of age was consistently 0%, while in the older dogs the results were 14.81%, 7.40%, 7.40% and 11.11% using the Rose Bengal plate test (RBPT), serum agglutination test (SAT), TAT and ELISA tests, respectively. All dogs up to 6 months of age were found to be seronegative because they were not sexually mature and active yet. Anyaoha (2015) revealed that in an earlier study in Nigeria, a seroprevalence of 3.4% in dogs younger than 1 year was reported, while a seroprevalence of 10.1% was reported in dogs 1–3 years old and 15.7% was reported in dogs above 3 years of age.

The findings recorded in the NMBM study area indicated a higher *B. canis* occurrence (9.8%) compared to the two other locations. The fact that NMBM had the highest average age of dogs (43.4 months), as compared to 29.9 months in the CJM-Gauteng and 40.6 months in the T&O, explains why NMBM had a higher risk of *B. canis* infection compared to the other two study areas. These findings affirm previous work of other researchers who concluded that the age of the dog, especially in sexually active dogs above 3 years of age, presents a greater risk for *B. canis* infection (Ayoola et al. 2016; Anyaoha 2015).

The higher the external parasite loads are on a dog, the greater will be its predisposition to *B. canis* occurrence (*p* = 0.001). In this work, the NMBM had the highest level of parasite infestation as well as the highest occurrence of *B. canis* in dogs. Certain factors may be responsible for heavy parasite loads in dogs, including but not limited to a lack of grooming for dogs, lack of ownership with consequent extensive freedom for roaming and poor parasite control using parasiticides. While no studies have directly associated external parasite infestation with *B. canis*, it is plausible that most dogs with heavy parasite infestations are unowned and may be free-roaming with higher risks of infection with *B. canis*. In addition, it is most likely that when controlling external parasites, other health risks inclusive of *B. canis* infection may be detected early and managed. It becomes necessary for authorities to consider the implementation of a comprehensive control programme that caters for both infectious diseases and parasite infestations.

Although ownership of dogs did not reach significance in the final analysis, in the univariate model it was observed that ownership of dogs was a positive predictor for the occurrence of *B. canis* (OR: 1684; *p* = 0.05). In addition, previous works have found that ownership of dogs predicted the occurrence of *B. canis*, in that the more stray dogs present, the higher the incidence of *B. canis* (Chikweto et al. 2013). However, these findings must be interpreted with caution because, while in former studies ‘ownership’ referred to individuals who presented their dogs at the clinics, or those who regularly provided medical care and management for their dogs, it is doubtful if the same definition can be applied in the present study. It should be noted that samples were obtained during the implementation of low-cost welfare services provided to the communities. In such instances, the economically impoverished individuals will more likely utilise those services as an opportunity to attend to the health of their animals. This may be a reason for the observed difference in this study compared to the others conducted previously.

## Conclusion

Although this study indicated dog age and, to a lesser extent, external parasite infection as the only risk factors for *B. canis* infection in dogs in the study area, based on the findings of the univariable analysis, other possible risk factors such as dog ownership and sterilisation need further investigation, especially in the informal sector of South Africa. The confirmation of the prevalence of *B. canis* in dogs in the study area has implications for humans. The prevalence of the disease in humans in South Africa is unknown and is in need of more attention and future research. *Brucella canis* is a zoonotic disease that affects children mostly and as such dog owners need to become more responsible and to be aware of the risk factors that contribute to possible *B. canis* infection in order to prevent and/or reduce human infections. Efforts from the veterinary authorities and welfare agencies should be intensified to reduce the number of free-roaming dogs in informal settlements. This will help minimise contact between infected dogs and the susceptible dog population. There is a need for public health authorities to educate the public on hygienic practices for people owning a pet dog, in particular children. Spaying or castration of dogs entering sexually maturity should be promoted to assist in reducing the burden of canine brucellosis.

## Appendix 1

**TABLE 1-A1:** Variables in the binomial multivariable logistic regression model.

Variable	B	SE	Wald	df	Sig.	Exp (B)
Age	−0.979	0.291	11.282	1	0.001*	0.376
External parasites	−1.109	0.340	10.659	1	0.001*	0.330
Ownership	0.521	0.397	1.727	1	0.189	1.684
Constant	−2.538	0.402	39.812	1	0.000	0.079

B, Bonferroni correction; SE, standard error; Df, degrees of freedom; Sig, significance; Exp, expressions.

* *p* < 0.05.
